# Optimal Design of Switchable Wearable Antenna Array for Wireless Sensor Networks

**DOI:** 10.3390/s20102795

**Published:** 2020-05-14

**Authors:** Łukasz Januszkiewicz, Paolo Di Barba, Sławomir Hausman

**Affiliations:** 1 Institute of Electronics, Lodz University of Technology, Wólczańska 211/215 Street, 93-005 Łódź, Poland; slawomir.hausman@p.lodz.pl; 2 Department of Electrical, Computer and Biomedical Engineering, University of Pavia, via Ferrata 5, 27100 Pavia, Italy; paolo.dibarba@unipv.it

**Keywords:** genetic algorithms, FDTD, finite-difference time-domain, wireless body area network, WBAN, signal-to-interference ratio, SIR, interference rejection, human body model, evolutionary computing, optimization, radiation pattern synthesis, switchable antenna

## Abstract

In the paper, we present a novel approach to the optimum design of wearable antenna arrays intended for off-body links of wireless body area networks. Specifically, we investigate a four-element array that has a switchable radiation pattern able to direct its higher gain towards a signal source and a lower gain towards an interference. The aim is to increase the signal to interference ratio. We apply a genetic algorithm to optimize both the spatial placement and the feed phasing of the elementary on-body antennas. We propose a simplified, computationally efficient model for the simulation of the array radiation pattern. The model is based on full-wave simulations obtained with a simplified cylindrical model of the human body. We also propose, implement, and evaluate four objective functions based on signal to interference ratio, i.e., min-max, nadir point distance maximization, utopia point distance minimization, and full Pareto-like. Our optimized design obtained with this approach exhibits a significant performance improvement in comparison to the initial heuristic design.

## 1. Introduction

Wireless sensor networks consist of small measurement devices that can transmit data using integrated wireless modules. Such arrangements can utilize wireless sensors that are located directly on the human body or are embedded in the clothes. This makes it possible to remotely monitor physiological parameters or vital signs of people as well as their motion, body posture, location, etc. Networks of such wearable wireless sensors can find numerous applications in healthcare, sports, or public safety because they unobtrusively gather data [1,2].

The critical feature of a wearable wireless sensor network is the size of the node that should be small enough not to limit the comfort of use. This requires small batteries that suffer from limited energy resources, which, as a consequence, reduces the maximum transmit power of the wearable nodes. For this reason, the power budget of the wireless link is very tight, and the separation of signal from interference is relatively small.

**Figure 1:**
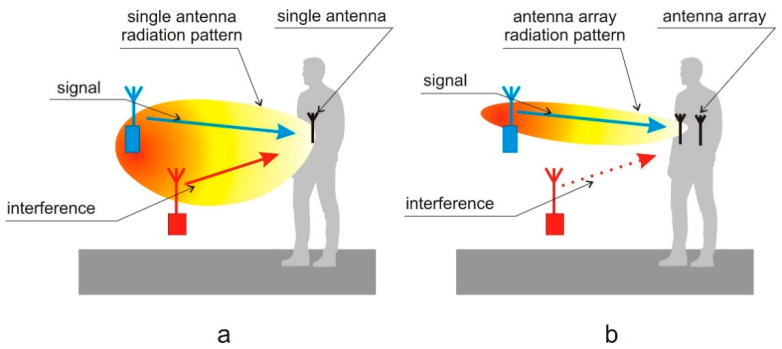
The principle of adaptive array operation for interference rejection: (**a**) single antenna realizes broad radiation pattern; (**b**) antenna array can direct the beam towards the signal.

Wireless sensor that works in the proximity of the human body utilizes an antenna that operates in the complex environment consisting of open space, human tissues, and clothes. The antenna radiation pattern that presents the angular distribution of gain is influenced by the body and is also sensitive to the placement of antenna [3]. In general, the antenna that is located close to the body (it is a so-called wearable antenna) has the radiation pattern with a wide main lobe, and its minima are directed towards the body. In such a case, for a wide range of angles, the antenna has almost constant gain. Wireless sensor networks that operate in the off-body mode transmit a signal between the remote transceiver that is located far from the body and the wearable sensor. When the wearable antenna radiation pattern has a wide main lobe, the useful signal, as well as the interference, can be received from different directions with a similar antenna gain and with similar power levels. Then, the signal-to-interference ratio (SIR) is low, which is unfavorable (see Figure 1a).

Several approaches to the improvement of SIR are known [4,5,6,7,8], mainly relying on optimum antenna design or simple switchable/reconfigurable antennas. However, to our best knowledge, on-body phased arrays have not been used to achieve this goal yet. A phased array antenna consists of a set of elementary antennas organized in a particular geometrical arrangement: linear, planar, circular, cylindrical, etc. The signal from the transceiver is connected through the combiner and feedlines to each elementary antenna. The radiation pattern of the array for a given number of elementary antennas and their spatial arrangement depends on the phase relationship at the antenna feed points, which follows from the phase shift in each elementary antenna feeding path. Signal-to-interference ratio (SIR) can be improved by forming the radiation pattern to direct the maximum gain of the array towards the signal and the minimum towards the interference [6,9] (see Figure 1b)—with constraints on the radiation pattern following from the array design. Phase switching can be automatically performed during system operation to improve the quality of transmission.

There have been numerous studies into the problem of circular antenna array optimization, e.g., [10,11,12,13,14,15,16]. In the case of uniform arrays [10,13,14], beamforming is controlled by the adjustment of amplitudes and phases in the feeding network of elementary antennas. Additionally, for non-uniform arrays [11,12,15,16] angular positions of the antennas can be adjusted, which provides even more degrees of freedom to the optimization algorithm and yields greater flexibility of the directivity pattern shaping. Among many algorithms successfully applied for antenna array optimization, particle swarm optimization [10,12,13] and genetic algorithms [11,14] are often used.

The goal of the optimization process is usually a single-objective improvement of array performance in terms of radiation pattern parameters (e.g., the side lobe level and the directivity). In [15] a single-objective design problem was reformulated as a multi-objective problem. It was possible to accomplish a set of non-dominated solutions and then indicate the best-compromise solution. Additionally, in [11] a multi-objective approach was presented to optimize the amplitude and phase of the excitation and the separation between the array elements. In our research, we also considered the optimization problem as initially single-objective and then bi-objective, showing that the latter approach is very effective in identifying a satisfactory solution.

In the literature, circular arrays that were the subject to the optimization process consist of elementary antennas that have omnidirectional radiation (in [10] an array of dipoles is used). In our study, we investigate the optimization of the array that consists of wearable antennas, which, to our best knowledge, is our original concept and was neither investigated nor published by other researchers.

In this paper, we consider the utilization of phased array antennas to improve the performance of the Narrow Band Internet of Things (NB-IoT) system that, due to the low power of transmitters, can be sensitive to interferences. In this case, the wearable phased antenna array is investigated that could be applied for the wireless system that monitors physiological parameters of people (Figure 1b). For simplicity, we assumed that this array consists of vertically oriented half-wave dipole antennas designed to operate in free space at 700 MHz frequency band which is allocated in Poland for the fifth generation of wireless communication systems that include NB-IoT. To simplify the feeding network of the wearable array, we assume that the phase shift of signal connected to the *l-th* antenna can take on one of two possible values $\alpha (l)\in \left\{0,\;\alpha_{const}\right\}$. In the prior studies, a wearable antenna array that was fed with no phasing was used to obtain the bi-directional radiation pattern [17]. It was shown that the radiation pattern of the array depends strongly on the placement of each antenna element. In the antenna array optimization process the position of individual radiators on the body is identified together with the value of phase shift $\alpha_{const}$ to improve the antenna SIR.

The paper is organized in the following way: after the Introduction, models of direct and inverse problems are formulated in Section 2, where the optimization approach is also presented; eventually, optimization results are presented and discussed in Section 3 and Section 4, respectively.

## 2. Models and Methods

### 2.1. Direct Problem Formulation

The direct problem can be formulated in the following way: “given antenna placement and phase shift values calculate the antenna radiation pattern.” For this purpose, we started our investigation with numerical models of wearable antenna array radiation patterns. We applied the Remcom XFdtd^®^ program that utilizes the finite-difference time-domain method (FDTD) [18]. It was used as the reference to elaborate the simplified analytical model of antenna array radiation pattern that was needed in the iterative optimization procedure.

**Figure 2:**
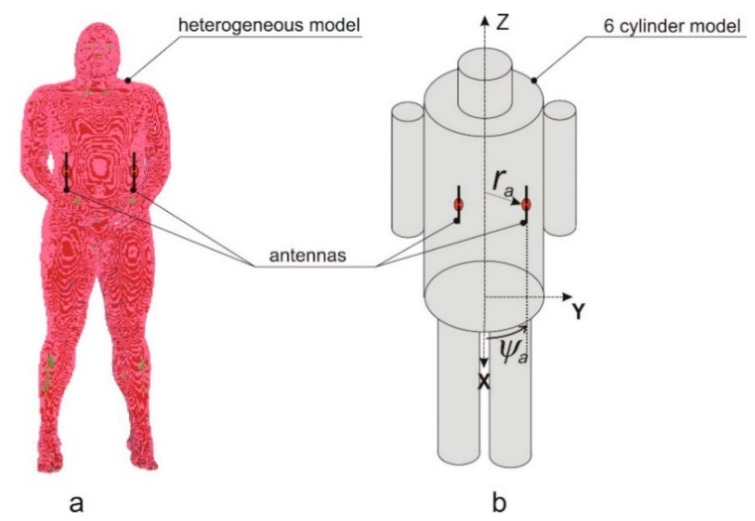
Antenna array on a human body model: (**a**) heterogeneous anthropomorphic model; (**b**) cylindrical model of the human body: *r_a_*, *ψ_a_*—coordinates of a single antenna.

The typical approach to computer simulation of wearable antenna radiation pattern uses numerical, heterogeneous models of the human body. In the Remcom XFdtd^®^ program, the heterogeneous model of the human body is available with different voxel sizes, from 1 to 10 mm. It represents the entire body, with its internal structure (see Figure 2a). We used this model with 5 mm voxel size for preliminary research on wearable an antenna array [17] and as a reference to the simplified models.

In this research, we aimed at optimizing the wearable antenna array by finding the optimum placement of elementary antennas on the body. This requires carrying out numerous simulations of array radiation pattern for different positions of the elementary antennas with respect to the body, preserving the constant distance of antennas to the body surface. In the case of anthropomorphic models, where the body shape is complex, maintaining a fixed distance between the antenna and body is difficult. For this reason, we utilized in Remocm XFDTD software the cylindrical body model presented in Figure 2b to investigate how the radiation pattern of the wearable antenna array depends on its geometry and phasing. This model consists of six cylinders that are made of tissue simulant material, and its usability for wireless body area network simulations was successfully verified in our previous research [19,20]. The radiation patterns of the wearable antennas obtained with the cylindrical model were in good agreement with the one obtained with the heterogeneous model [21]. The cylindrical model is convenient for application to WBAN optimization when the antenna position is variable, but its distance to the body surface is fixed [22,23]. In this case, the position of the antenna can be controlled easily with two coordinates *r_a_*, *ψ_a_* in a cylindrical coordinate system presented in Figure 2b.

**Figure 3:**
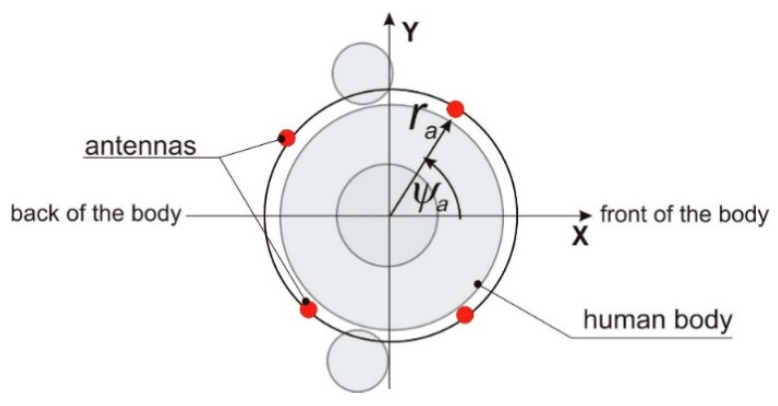
Wearable antenna array—simplified geometry of four-element planar circular array.

For an iterative optimization, the numerical burden of simulation with body model and Remcom^®^ XFdtd program is relatively high because the antenna radiation pattern for each combination of phasing is obtained with simulations which take a few minutes each. We propose a simplified, analytical model of a wearable antenna array to overcome this limitation. This approach assumes that the considered wearable antenna array is a planar circular i.e., each antenna is located on a surface of a cylindrical model of the trunk, and its *z* coordinate is equal to 1.3 m above the feet. The simplified geometry of the antenna array is presented in Figure 3. This planar circular array is formed by vertical dipoles placed in a fixed distance from the body (*r_a_* = 167 mm), and each dipole position is defined by *ψ_a_* angle $G_{a}\left(\varphi \right)$.

The radiation pattern of a single wearable antenna depends on the antenna position towards the body. The maximum value of antenna gain is directed in the direction that is opposite to the trunk. To model this feature, we proposed an analytical formula to calculate the gain $G_{a}(\varphi )$ in the horizontal *x-y* plane of the wearable dipole, which simultaneously depends on antenna position angle *ψ_a_* defined in Figure 2b. The directivity pattern of the elementary antenna is modeled by Equation (1), which is a heuristic approximation proposed by the authors.

(1) $$G_{a}\left(\varphi ,\psi_{a}\right)=G_{0}\cdot cos^{4}\left(\frac{\varphi -\psi_{a}}{2}\right)$$

where φ∈[0, 2π) is the coordinate in the cylindrical system and $G_{0}$ is a scaling parameter.

**Figure 4:**
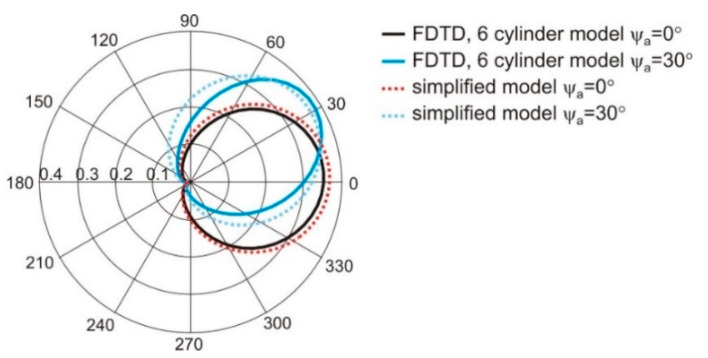
The radiation pattern of a single wearable antenna obtained with the simplified model given in Equation (1) compared to the results of simulations with the FDTD method and cylindrical model of the body.

The $G_{0}$ can be used to scale the maximum value of the dipole gain obtained from Equation (1). The proximity of the human body deteriorates the gain of a simple dipole antenna that was used in our research. In this case, the distance of the dipoles to the body model was equal to 10 mm, and the maximum value of dipole gain in the linear scale was reduced to 0.38. To obtain the best matching of gain calculated with Equation (1) and values obtained from finite difference time domain based (FDTD) simulations with cylindrical model the value of $G_{0}$ = 0.35 was applied. In Figure 4, the radiation pattern of a single dipole antenna located on body that was obtained with the simplified model given in Equation (1) is compared with results of simulations with FDTD method and cylindrical model of the body for two values of antenna position angle *ψ_a_* equal to 0° and 30°. The results obtained with the use of one and the other model are similar. The angular distribution of antenna gain is similar in both cases. Additionally, the effect of changing the direction of maximum radiation (angle for the maximum value of gain) depending on antenna position *ψ_a_* is preserved in the simplified analytical model.

Having the formula that approximates radiation of the single antenna $G_{a}\left(\varphi ,\psi_{a}\right)$ we calculated the gain of the four-element circular array $G\left(\varphi ,\psi_{a}\right)$ given by Equation (2) [24]:

(2) $$G\left(\varphi ,\psi_{a}\right)=\overset{4}{\underset{l=1}{\sum }}G_{a}\left(\varphi ,\psi_{a}\left(l\right)\right)e^{jkr_{a}\cos\left(\varphi -\psi_{a}\left(l\right)+\alpha (l)\right)}$$

where:

φ∈[0, 2π), *k—*wave number, *l—*radiator number, *r_a_* = 167 mm—array radius, α(l)*—*feeding phase shift of the *l*-th antenna, *ψ_a_*(*l*) angular position of the *l*-th antenna.

**Figure 5:**
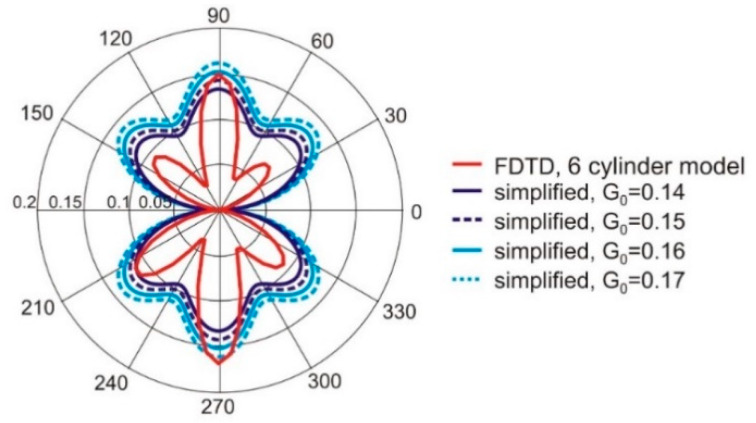
The radiation pattern of the wearable antenna array obtained with FDTD based simulations and the cylindrical model of the body compared to results obtained with the simplified model given by Equation (2) for various values of *G_0_.*

The radiation pattern of antenna array obtained with Equation (2) was compared with the results of simulations with the cylindrical model of the wearable array in FDTD. In Figure 5 the radiation pattern of the four-element antenna array obtained with simulations in the XFdtd program and cylindrical body model are compared to the results obtained with the simplified method. The angular positions of the antennas *ψ_a_* are: −20°, 20°, 160°, 200°. The simplified model (Equation (2)) was examined with different values of *G_0_* parameter. The best results were obtained for *G_0_* = 0.16. The angular distributions of antenna gain obtained with FDTD and Equation (2) are similar, but the simulation time was significantly shorter in the case of the simplified model given by Equations (1) and (2).

**Figure 6:**
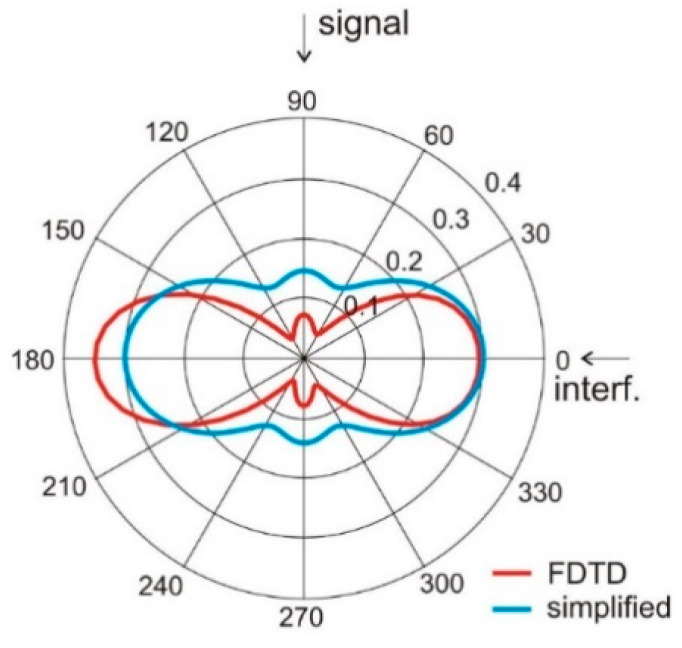
Antenna array radiation pattern *G*(*φ*) with four elements located on the *ψ_a_*: [−20°, 20°, 160°, 200°], phasing vector α = [0,0,0,0].

**Figure 7:**
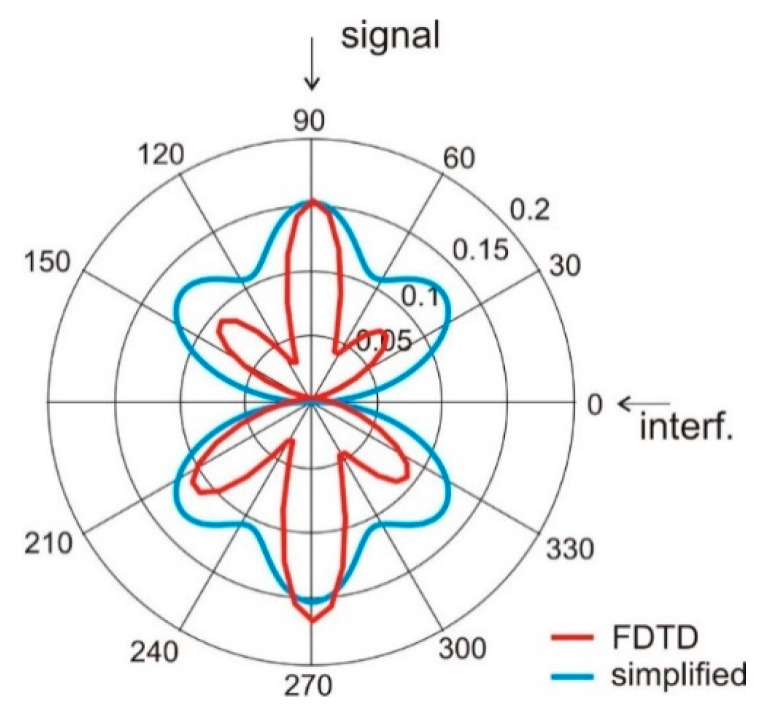
Antenna array radiation pattern *G*(*φ*) with four elements located on the *ψ* = [−20°, 20°, 160°, 200°]; α = [0, π, π, 0].

Having the simplified model given in Equation (2), we examined the ability of the proposed four-element wearable array to form its radiation pattern for interference rejection. In this experiment, we assumed the angular positions of the antennas *ψ_a_* are: −20°, 20°, 160°, 200° and the signal comes from direction *φ_s_ =* 90° while the interference is located at *φ_i_ =* 0°. For such a case, presented in Figure 6, the array with phasing vector *α* = [0°,0°,0°,0°] direct the main beam with the greatest value of gain towards interference and the minimum value of gain towards the signal. The radiation pattern of this array with phasing vector *α* = [0°, π, π, 0°] has the radiation pattern presented in Figure 7. Here, the greatest value of gain is directed towards signal and the gain minimum towards interferences. This illustrates the ability of our wearable antenna array to increase the signal to interference ratio.

The results presented in Figure 6 and Figure 7 show that both simplified model and FDTD simulations with the cylindrical model result in the same angular positions of gain minima and maxima. This suggests the possibility of using the analytical model (Equations (1) and (2)) in the optimization process.

### 2.2. Inverse Problem Formulation

We formulate the inverse problem in the following way: “Find the position of array elements and phase shift values such that the highest value of radiation pattern is directed towards signal while the lowest value towards interferences, for all the considered positions of signal and interference.” To this end, an optimization problem is set up, the degrees of freedom of which are the angular position vector *ψ*(*1)*, *ψ*(*2)*, *ψ*(*3*), *ψ*(*4*) of the antennas composing the four-element circular array and the value of non-zero phase shift $\alpha_{const}$ that can be applied in the feeding network; so, a five-dimensional design space is considered. Accordingly, a possible objective function *f* to be maximized should express the SIR which is the ratio of the array gain towards the signal *G_s_* to the array gain towards the interference *G_i_* that could be obtained against all possible combinations of *n-th* antenna feeding phases, *n* = [1.4], given the positions of signal and interference. For this reason, the radiation pattern of the array has to be calculated for *P* combinations of antenna phasing vector, to obtain the objective function components *G_s_* and *G_i_*, namely

$$P=\left(2^{4}-1\right)=15$$

This computation has to be performed for each signal position *φ_s_* and interference position *φ_i_*. We consider *S* combinations of signal and interference angles separated by 45° in a 360° angle, namely

$$S=\left(2^{\frac{360{}^{\circ}}{45{}^{\circ}}}-8\right)=248$$

The number of calls to the radiation pattern computations at each iteration of an optimization algorithm is a reasonable estimate of the cost per iteration *C* that can be calculated as:

C=S*P=3720

**Figure 8:**
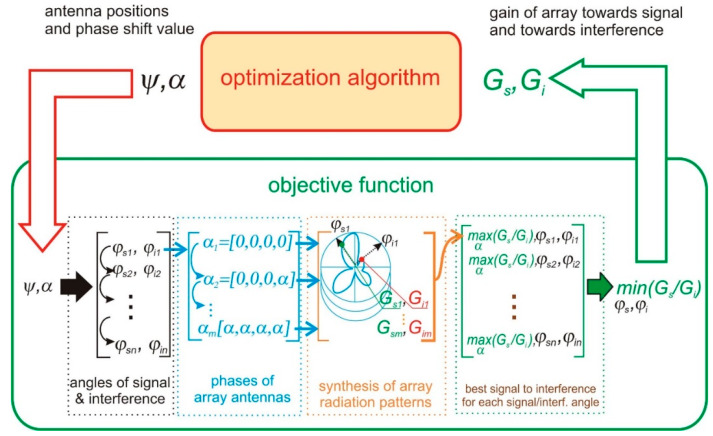
Optimization loop flow-chart showing the algorithm for the calculation of objective function components.

In other words, the total number of radiation pattern computations depends on the number of optimization algorithm iterations—3720 in every single iteration. For this reason, the use of a cost-effective analytical model of array radiation pattern like, e.g., Equation (2) is mandatory because performing 3720 simulations based on FDTD in each optimization step would be impractical due to the computational time. The conceptual flow-chart presenting the algorithm for the calculation of objective function components is shown in Figure 8.

In the following sections, we present four diverse versions of the objective function, which we proposed and examined. This approach follows from our experience that for complex problems in electromagnetics, the capability of stochastic optimization methods for an effective exploration and exploitation of the objective space strongly depends on the objective function formulation. We intended to identify a problem-specific function that offers the fastest convergence.

#### 2.2.1. Min-Max Approach

The first approach to solve the inverse problem formulated in Section 2.2 was based on a single-objective evolutionary algorithm of the lowest order—EStra, originally proposed in [25]. The objective function *f_m_* defined by Equation (3), to be maximized, expresses the min-max norm of the ratio of the array gain towards the signal to the array gain towards the interference (SIR) that could be obtained with all possible combinations of *n-th* antenna phases α(n) *n* = [1.4] for the $\psi_{a}$ vector dictated by the optimization algorithm, namely

$$f_{m}\left(\psi_{a},\;\alpha (n)\right)=\underset{\varphi_{s}\varphi_{i}}{\min}\left|\underset{\alpha \left(n\right)}{\max}\frac{G_{s}\left(\psi_{a},\varphi_{s},\;\alpha (n)\right)}{G_{i}\left(\psi_{a},\varphi_{i},\;\alpha (n)\right)}\right|;$$

*φ_s_*=0°, 45°, … 315°; *φ_i_* = 0°, 45°, … 315°; *φ_s_* ≠ *φ_i_;*

(3) $$\alpha \left(n\right)=0{}^{\circ},\;\alpha_{const}$$

Therefore, maximizing Equation (3) gives rise to a single-objective optimization. Nevertheless, the following remark can be put forward. After a few numerical experiments, it is straightforward to realize that the objective function components *G_s_* and *G_i_* defined in Equation (3) are competing objectives. In fact, if *G_s_* and *G_i_* are independently considered, it comes out that the values of antenna positions *ψ_a_* and phase angle *α* which maximize *G_s_* function alone are not the same which minimize *G_i_* function alone. This puts the ground for the multi-objective approach developed in the subsequent sections.

#### 2.2.2. Nadir Point Distance Maximization

As an alternative to the min-max approach, in this subsection and in the next one we propose two other single-objective formulations amenable to a concept of multi-objective optimization theory. In fact, objective function components *G_s_* and *G_i_* define a 2D objective space where the Pareto front, i.e., the set of solutions exhibiting the best trade-off between competing objectives *G_s_* and *G_i_,* takes place. In particular, two reference points can always be found in a straightforward way in a 2D objective space:

- utopia *U*, i.e., the point the coordinates of which are the best values of each objective (in our case, *U_s_* is the maximum of *G_s_* function, independently considered and subject to the problem constraints; a similar explanation holds for *U_i_*);
- nadir *R*, i.e., the point the coordinates of which are the worst values of each objective.

**Figure 9:**
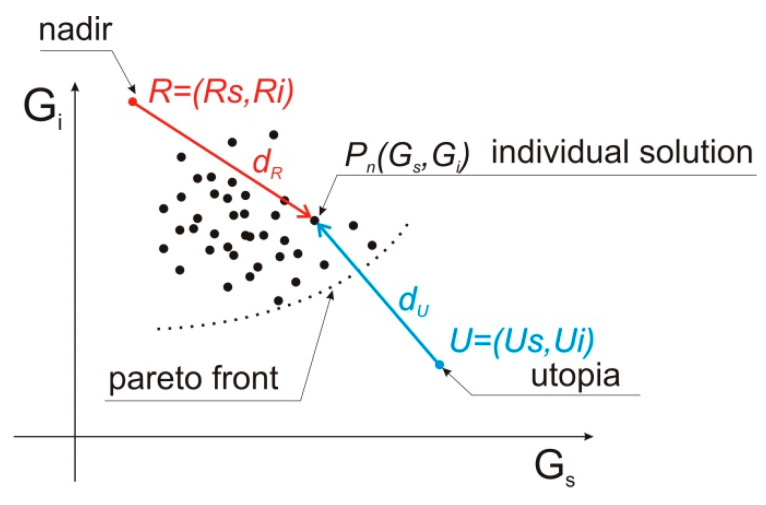
The definition of *d_R_* and *d_U_* in the objective function components space.

According to a geometric interpretation, utopia and nadir are located at the independent vertices of a rectangle which incorporates the Pareto front (Figure 9). Put simply, “good” solution points are those located within the rectangle, far from nadir point and close to utopia point.

Moving from this background, we decided to implement a single-objective optimization inspired by the utopia-nadir concept, with the final aim of approximating a solution located along the front of best compromises. Accordingly, the second proposed approach utilizes the concept of distance of individual solution point to nadir point, which is defined in the objective space as shown in Figure 9. As the first step, we approximated the values of utopia coordinates and nadir coordinates; then, we defined the objective function *f_r_* defined by Equation (4), to be maximized, which expresses the *d_R_* distance from nadir point in objective function components space. Therefore, *f_R_* is a scalar preference function conceived in a bi-objective context. The coordinates of nadir point are (*R_s_*,*R_i_*) = (0.1,0.25). $f_{r}\left(\psi_{a},\;\alpha (n)\right)=\underset{\varphi_{s},\varphi_{i}}{\min}\left|\underset{\alpha \left(n\right)}{\max}\;d_{r}\right|$;

$$d_{r}=\sqrt{{\left(G_{s}-R_{s}\right)}^{2}+{\left(G_{i}-R_{i}\right)}^{2}}$$

*φ_s_* = 0°, 45°, … 315°; *φ_i_* = 0°, 45°, … 315°; *φ_s_* ≠ *φ_i_;*

(4) $$\alpha (n)=0{}^{\circ},\;\alpha_{const}$$

#### 2.2.3. Utopia Point Distance Minimization

The third proposed approach utilizes the concept of distance of individual solution point to utopia point, which is defined in the objective space as shown in Figure 9. To this end we defined the objective function *f_u_* by means of Equation (4), to be minimized, which expresses the *d_U_* distance from nadir point in objective function components space. The coordinates of nadir point are (*U_s_*,*U_i_*) = (0.25,0.15).

$$f_{u}\left(\psi_{a},\;\alpha \left(n\right)\right)=\underset{\varphi_{s,}\varphi_{i}}{\max}\left|\underset{\alpha \left(n\right)}{\min}\;d_{u}\right|;$$

$$d_{u}=\sqrt{{\left(G_{s}-U_{s}\right)}^{2}+{\left(G_{i}-U_{i}\right)}^{2}}$$

*φ_s_* = 0°, 45°, … 315°; *φ_i_* = 0°, 45°, … 315°; *φ_s_* ≠ *φ_i_;*

(5) $$\alpha (n)=0{}^{\circ},\;\alpha_{const}$$

#### 2.2.4. Full Pareto-Like Optimization: P-EStra

Eventually, a full multi-objective approach in terms of (*G_s_*, *G_i_*) space was considered, without resorting to a scalar preference function like in previous Section 2.2.2 and Section 2.2.3.

To this end, an algorithm based on a multiobjective (1+1)-evolution strategy (P-EStra) inspired by Pareto optimality theory is used [25,26]. The algorithm is implemented in such a way that a new design vector *x = m + du* (offspring) is accepted if and only if *x* dominates the current design vector *m* (parent) according to Pareto definition for *m* ≥ 2 objective functions, subject to problem constraints. This means that the offspring is accepted only if it simultaneously improves all the objectives. In turn, d is the standard-deviation vector associated to *m*, while u∈[0,1] is a normally distributed perturbation. Vector d is initialized as *d_o_*_,_ and the value of its elements is proportional to the feasible range of the corresponding design variable. Vector *d*, which drives the search, is updated according to the prescribed rate of success in improving the objective functions; that is where the self-adaptation of the strategy parameter comes in: *d* itself undergoes a modification, which is ruled by a randomized process. In fact, given the correction rate q∈(0,1), considering the *k*-th iteration, $d_{k+1}=q^{-1}\cdot d_{k}$ (or $d_{k+1}=q\cdot d_{k}$) is set to force a larger (or smaller) standard deviation of Gaussian distribution associated with *x* in the next iteration. In other words, the solution vector x and the standard-deviation vector *d* are both subject to random mutation. In a basic (1+1) implementation, the operator of Paretian selection allows for the best individual, out of parent *m* and offspring *x*, to survive to the next generation. In other words, an offspring individual is selected to survive if and only if it is better, or at least non-worse, than the parent individual against all the m objective functions. This way, given an initial point, there is a non-zero probability that the optimization trajectory eventually leads to a point belonging to the Pareto optimal front. The algorithm converges when the ratio of the largest value of d vector elements to the corresponding element of the initial standard-deviation vector *d_o_* is smaller than the prescribed search tolerance.

The basic computational cost c of P-EStra algorithm can be estimated as

$$c\approx c_{0}\cdot n_{i}\cdot n_{p}\cdot n_{f}$$

where *c_0_* is the hardware-dependent time necessary to run a single solution of the direct problem associated to the optimization problem (in our case, the computation of directivity pattern of antenna array), *n_i_* is the number of convergence iterations for a prescribed search accuracy, *n_p_* is the number of evolving solutions (in our case, *n_p_* = 1), and *n_f_* is the number of objectives.

In principle, there is no limitation to the number of objectives the algorithm can process, and this is a potential advantage for a high-dimensionality problem. Summing up, the evolutionary algorithm starts from a guess solution, which can be either user-supplied or randomly generated, iteratively originates a search trajectory driven by the concept of non-dominated solution, and eventually converges to a Pareto-optimal solution.

## 3. Results

The optimization process was performed subject to constraints on the possible positions of the four elementary antennas following from the user’s clothes design. The azimuthal coordinates of the antennas *ψ_a_* were limited to the range from −60° to −10° for antenna 1, 10° to 60° for antenna 2, 120° to 160° for antenna 3, and 200° to 240° for antenna 4. The initial positions of antennas were *ψ_a_* = [−20°, 20°, 158°, 202°]. The allowable value of phase-shift $\alpha_{const}$ was within the range from 45° to 270° and its initial value was 90°. In this case, the initial value of objective function *f* was equal to 0.84.

**Figure 10:**
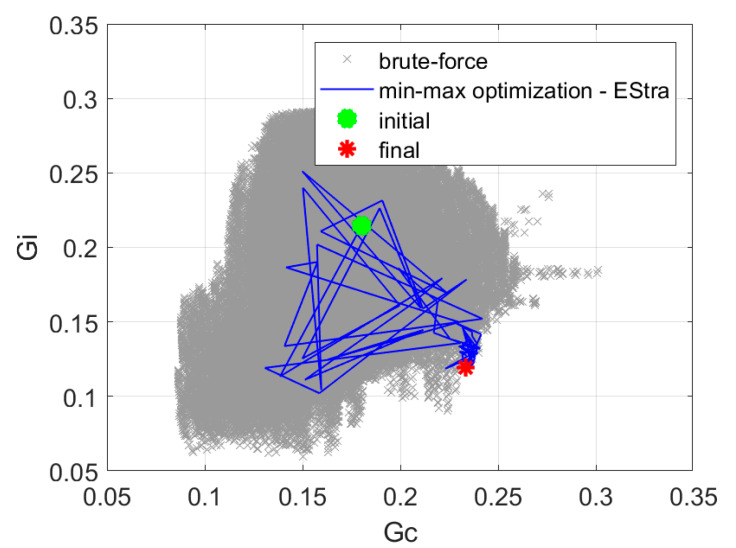
The history of optimization in the space of objective function components for min-max optimization.

**Figure 11:**
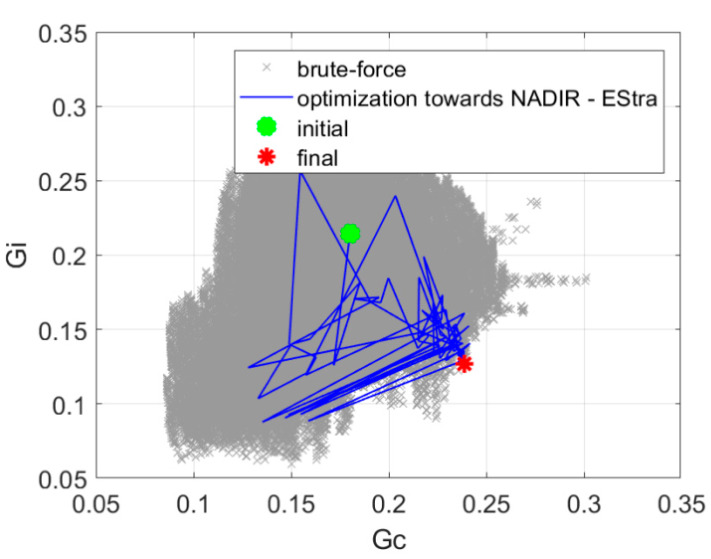
The history of optimization in the space of objective function components for optimization with nadir point distance maximization.

**Figure 12:**
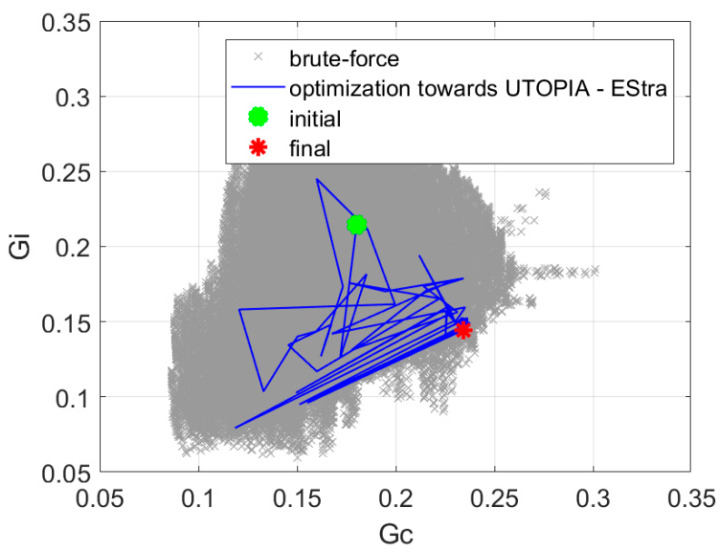
The history of optimization in the space of objective function components for optimization with utopia point distance minimization.

**Figure 13:**
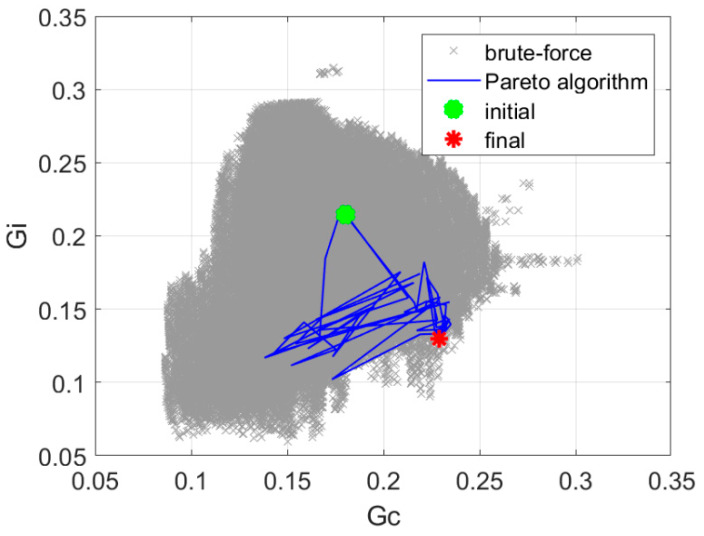
The history of optimization in the space of objective function components for optimization with P-EStra algorithm.

**Figure 14:**
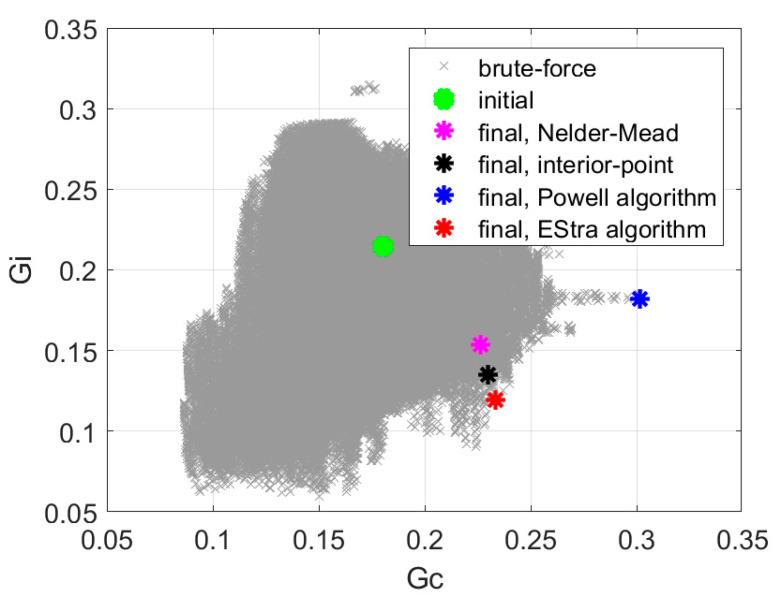
Solutions obtained for the single-objective min-max formulation with the Nelder–Mead, “interior-point”, Powell, and EStra algorithms, respectively.

Prior to the optimization, we made a brute-force exploration of the design space. The simplified numerical model that we applied in our research (Equation (2)) is very effective in terms of numerical burden required for the simulation. Therefore, it was possible to explore the entire design space of five parameters with 5° steps that gave c.a. 450,000 points within a few hours. The results of this exploration, once mapped in the objective space, are presented in Figure 10, Figure 11, Figure 12, Figure 13 and Figure 14 in grey color. Although the exploration points have been randomly generated according to uniform probability density, it can be noted that the corresponding distribution in the objective space is not really uniform, being characterized by a few peninsula-shaped branches. This is because functions *G_s_* and *G_i_* are nonlinear functions of the design variables, and nonlinearity determines a non-uniform distribution of points.

The min-max approach presented in Section 2.2.1 resulted in 109 iterations, and the value of the objective function was increased to 1.96. The final positions of the antennas are *φ_a_* = [−44.9°, 45.7°, 134.8°, 225.4°], and the phase shift value was $\alpha_{const}$ = 117.2°. The optimization process required 7 min of computation time on a PC computer equipped with Intel i7 processor. Figure 10 presents the history of optimization in the objective function components space as well as the results of a brute-force exploration of the design space.

The nadir point distance maximization presented in Section 2.2.2 resulted in 145 iterations, and the value of the objective function was increased to 1.87. The optimization process required 10 min of computation time on a PC computer equipped with Intel i7 processor. Figure 11 presents the history of optimization in the objective function component space. The results of a brute-force search of the design space are also presented.

The utopia point distance minimization presented in Section 2.2.3 resulted in 71 iterations, and the value of the objective function was increased to 1.62. The optimization process required 5 min of computation time on a PC computer equipped with Intel i7 processor. Figure 12 presents the history of optimization in the space of objective function components. The results of a brute-force search of the design space are also presented.

The Pareto-optimization presented in Section 2.2.4 resulted in 56 iterations, and the value of the objective function was increased to 1.76. The optimization process required 3 min of computation time on a PC computer equipped with Intel i7 processor. Figure 13 presents the history of optimization in the space of objective function components. The results of a brute-force search of the design space are also presented. The results of the comparison of optimization algorithm variants in terms of performance are presented in Table 1.

Recognizing the fact that we achieved the best solution using the single-objective min-max approach, we tested some other representative optimization strategies, namely Nelder–Mead [27,28], “interior-point” [29,30,31], and Powell [32]. Comparison of convergence and objective function value for these algorithms using the single-objective min-max formulation is presented in Table 2. Solutions in the space of objective function components are shown in Figure 14. The single objective EStra algorithm needed 109 iterations, and the value of the objective function was increased from 0.84 to 1.96. The “interior-point” algorithm implemented in Matlab function *fmincon* identified the solution with the objective function value of 1.7 with 628 iterations. The Nelder–Mead algorithm implemented in Matlab *fminsearch* function found the value of the objective function equal to 1.47 in 445 iterations. The value of the objective function equal to 1.66 was found by the Powell algorithm with 415 iterations.

## 4. Discussion

**Figure 15:**
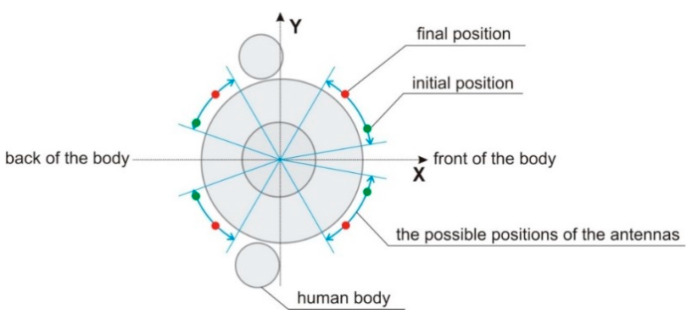
Elementary-antenna placement optimization. The initial and optimized (final) positions are shown.

**Figure 16:**
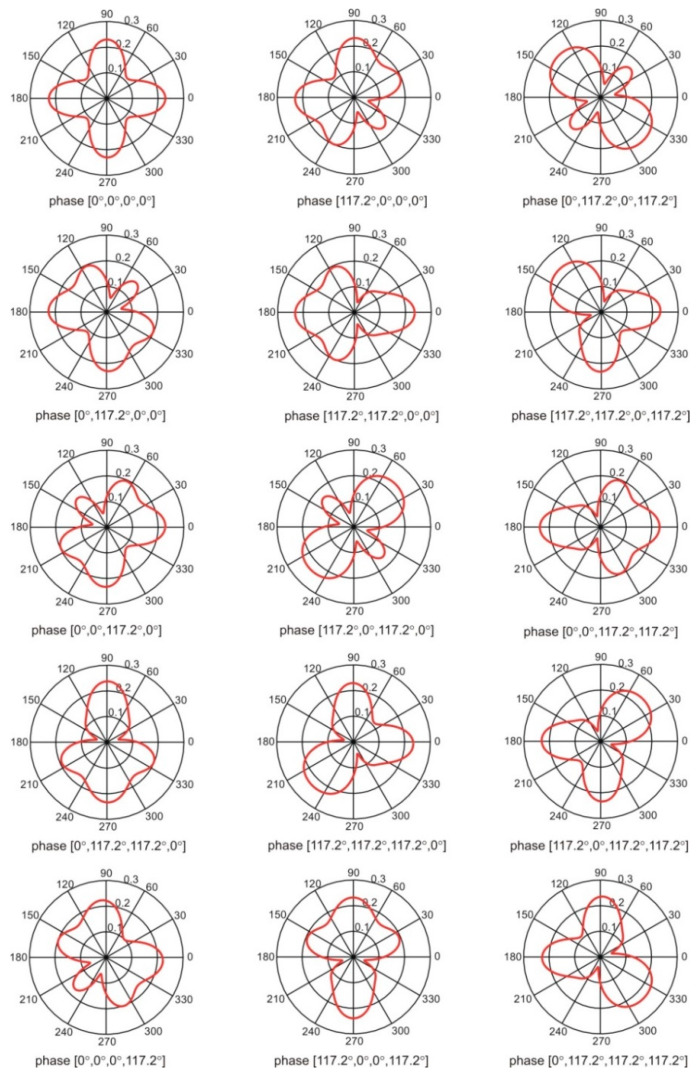
The radiation patterns of the optimized antenna array for all combinations of phasing.

In our investigation, we used four optimization approaches (min-max, nadir point distance maximization, utopia point distance minimization, and full Pareto-like) to the antenna array performance optimization, as described in Section 2.2. Out of the four approaches, we obtained the best results using the min-max version of the optimization algorithm. In Figure 15 we show the result of the elementary-antenna placement optimization. The initial and optimized (final) positions are presented for comparison. It can be observed that the initial and final positions differ significantly, but the bilateral symmetry of placement is maintained. It is worth noting that the symmetry, although intuitively correct, was not a-priori forced by the algorithm. Under the circumstances, the fact that our stochastic optimization converged to the symmetry can be regarded as a manifestation of its proper operation. The resultant optimized array has switchable directivity with 15 possible patterns following from all possible combinations of phase shift values (0 or $\alpha_{const}=117.2)$ fed to the elementary antennas. All 15 possible patterns are illustrated in Figure 16. It can be concluded that even for such a small four-element array, the variability of the null and lobe positions of the patterns offer a possibility to improve the signal-to-interference ratio significantly, provided that the source of the interference and the signal are angularly spaced by 45° or in most cases even only ca 30° (see Figure 16).

**Figure 17:**
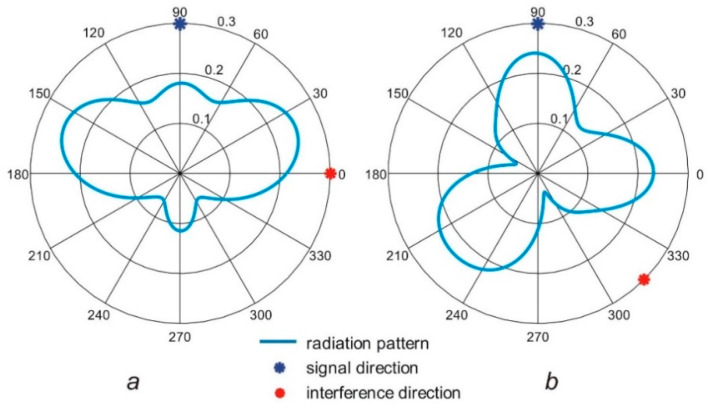
The radiation patterns of the antenna array for the worst combination of signal and interference angles: (**a**) before optimization, (**b**) after optimization.

Example radiation patterns of the antenna array before and after the optimization process with min-max version of the algorithm are presented in Figure 17. For the initial antenna configuration, the worst case was with the useful signal located at *φ_s_* = 90° and the interference located at *φ_i_* = 0°. The phasing vector [90° 0° 0° 90°] provided the SIR value equal to 0.84. For the optimized array, the worst case was for the signal located at *φ_s_* = 90° and interference located at *φ_i_* = 315°. In this case the phasing vector [117.2° 117.2° 117.2° 0°] yields the value of SIR = 1.96.

**Figure 18:**
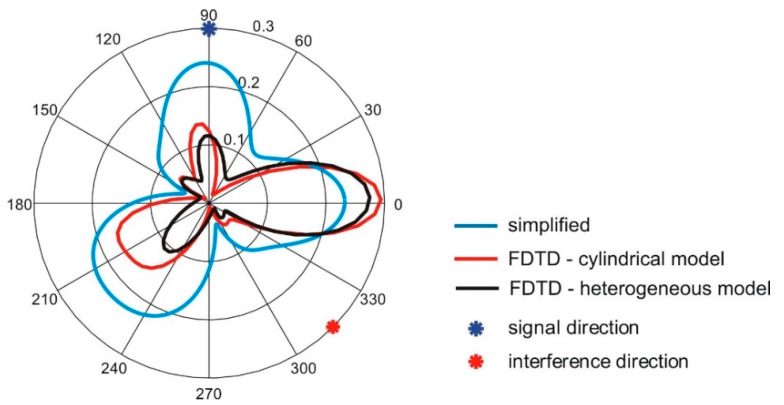
The radiation patterns of the optimized antenna array for the worst combination of signal and interference angles after optimization, obtained with the simplified model, cylindrical model, and heterogeneous model.

Figure 18 presents the radiation patterns of the optimized antenna array for the worst combination of signal and interference angles after optimization, obtained with the simplified model, cylindrical model, and heterogeneous model. The angular distributions of antenna gain obtained with the FDTD and simplified model used in the optimization process are similar, but the values of gain are different.

In the case of min-max optimization, the algorithm required 109 iterations to find the final solution, and the number of calls to the radiation pattern computations was equal to 405,480. Thanks to the computationally efficient simplified model of the wearable antenna array radiation pattern used here, the final solution was found in 7 min. The time that would be needed for the optimization with the FDTD simulations of radiation pattern (which takes one minute each) would be approximately 280 days, which can be considered prohibitive.

## 5. Conclusions

In our study, we considered a wearable four-antenna phased array synthesizing a switchable directivity pattern intended to improve the signal to interference ratio in off-body links. We applied an evolutionary algorithm to optimize the spatial placement and feed phasing of the elementary on-body antennas. To simplify the future implementations, we assumed only two possible values of phase (0 or $\alpha_{const}$) in the antenna feeding network. At this stage of our research, for simplicity, we considered an array consisting of half-wave dipoles with vertical orientation on the body. Consequently, we optimized five variables: four angular positions of the elementary antennas on the body and a single value of the phase shift $\alpha_{const}$. In each iteration of the optimization loop, we calculated the signal-to-interference ratio for 3720 combinations of signal and interference angles of arrival and feed phasing vectors. This number of full-wave electromagnetic simulations would be prohibitive. Therefore, to keep the computation time realistic, we applied a considerably simplified model of the wearable antenna array. We proposed and tested four diverse versions of the objective function, i.e., min-max, nadir point distance maximization, utopia point distance minimization, and full Pareto-like. Our experience justifies the assumption that for complex problems in electromagnetics, the capability of stochastic optimization methods for an effective exploration and exploitation of the objective space depends strongly on the objective function formulation. The common denominator linking the diverse formulations was the same computational method, i.e., an algorithm of evolutionary computing in either its single-objective version (min-max, nadir point, utopia point formulations) or multi-objective version (full Pareto-like formulation). Out of the four versions, we obtained the best results using the min-max objective function with a single-objective evolutionary algorithm of the lowest order—EStra. The single-objective optimization performed with three other algorithms: Nelder–Mead, Powell and “interior-point” not only identified inferior solutions but also with significantly greater number of iterations; moreover, the solutions independently identified by EStra and Powell are mutually non-dominated, but EStra solution was found in a substantially shorter runtime. In fact, the fastest convergence was observed with Pareto-like optimization that required approximately half of the number of calls to the objective function compared to min-max algorithm, giving a solution that was similarly good. Our investigation indicates that evolutionary optimization algorithms can be successfully applied to significantly improve the design of wearable antenna arrays in wireless body area networks. The other way around, i.e., comparing various optimization algorithms belonging to either the deterministic class or the stochastic one, against a specific formulation of the inverse problem, would be surely meaningful. Nevertheless, the approach would imply a substantial number of experiments: in the basic case of only two algorithms against four formulations, 2^4^ optimization runs would be in order; for the time being, this was out of the scope of our research.

In our follow-up research, we will investigate simplified but more accurate analytical models of wearable antenna array radiation patterns taking into account the interaction with human arms as well as coupling between the antennas. We will also investigate modeling arrays consisting of antennas that are designed to operate in proximity of the human body using our experience gathered with half-wave dipoles.

## Figures and Tables

**Table 1:** Comparison of optimization algorithm variants in terms of performance.

Variant of the Optimization Algorithm	Objective Function Value	Number of Iterations
Min-max	f_m_ = 1.96	109
Nadir point distance maximization	f_r_ = 1.87	145
Utopia point distance minimization	f_u_ = 1.62	71
Pareto-like optimization	f_p_ = 1.76	56

**Table 2:** Comparison of convergence and objective function value for several optimization algorithms.

Algorithm	Number of Iterations	Objective Function Value
EStra	109	1.96
Nelder–Mead simplex (Matlab function *fminsearch*)	445	1.47
Interior-point (Matlab function *fmincon*)	628	1.70
Powell	415	1.66
